# Ionizing radiation alters organoid forming potential and replenishment rate in a dose/dose-rate dependent manner

**DOI:** 10.1093/jrr/rrab120

**Published:** 2021-12-31

**Authors:** Yuki Fujimichi, Kensuke Otsuka, Masanori Tomita, Toshiyasu Iwasaki

**Affiliations:** Sustainable System Research Laboratory, Central Research Institute of Electric Power Industry, 2-11-1 Iwado kita, Komae-shi, Tokyo 201-8511 Japan; Sustainable System Research Laboratory, Central Research Institute of Electric Power Industry, 2-11-1 Iwado kita, Komae-shi, Tokyo 201-8511 Japan; Sustainable System Research Laboratory, Central Research Institute of Electric Power Industry, 2-11-1 Iwado kita, Komae-shi, Tokyo 201-8511 Japan; Sustainable System Research Laboratory, Central Research Institute of Electric Power Industry, 2-11-1 Iwado kita, Komae-shi, Tokyo 201-8511 Japan

**Keywords:** stem cells, Lgr5, organoids, small intestine, large intestine, dose-rate

## Abstract

Intestinal organoids are an *in vitro* cultured tissue model generated from intestinal stem cells, and they contain a mixture of epithelial cell types. We previously established an efficient ‘one cell/well’ sorting method, and defined organoid-forming potential (OFP) as a useful index to evaluate the stemness of individual cells. In this study, we assessed the response to radiation dose and dose-rate by measuring both OFP and the percentage of stem cells in the crypts. After high-dose-rate (HDR, 0.5 Gy/min) irradiation *in vivo*, the percentage of stem cells in the harvested crypt cells decreased, and the replenishment of cycling stem cells originating from dormant cells was enhanced, but OFP increased in cells irradiated with a total dose of >1 Gy. In contrast, at a total dose of 0.1 Gy the percentage of stem cells reduced slightly, but neither replenishment rate nor OFP changed. Furthermore, the response to 1 Gy of low-dose-rate (LDR) irradiation was similar to the response to 0.1 Gy HDR irradiation. These results suggest that 0.1 Gy HDR irradiation or 1 Gy LDR irradiation does not alter stemness. Additionally, the OFP increase in the colon in response to irradiation was smaller than that in the duodenum, similar to the percentage of stem cells. Understanding the differences in the response of stem cells between the colon and the duodenum to radiation is important to clarify the mechanisms underlying the development of radiation-associated intestinal cancers.

## INTRODUCTION

The intestinal tract is highly susceptible to developing radiation-induced cancer [1], the origin of which has been identified as intestinal stem cells [2]. The intestinal response to radiation at high-doses (HDs) and high-dose-rates (HDRs) has been extensively studied for medical purposes [3]. However, understanding the response to low-doses (LDs) and low-dose-rates (LDRs) is more relevant for assessing the health effects of radiation exposure in radiological workers and the general public. It remains unclear whether LD and LDR irradiation can lead to intestinal carcinogenesis. Therefore, studies on the biological mechanisms of radiation-associated intestinal cancer are urgently needed. Specifically, the evaluation of the intestinal radiation response in terms of dose/dose-rates will inform regulations for scientifically reasonable radiation protection against LD and LDR.

The small intestine consists of the duodenum (the proximal small intestine), jejunum and ileum, whereas the large intestine consists of the cecum, colon and rectum. Histologically, the small intestinal lumen is lined by villi and the crypts are located under the villi in the depths of the intestinal lining. The colon has crypt-like structures, but no villi. The intestinal stem cells are located in the bottom of the crypts. These cells are either actively or slowly cycling, and are characterized by their corresponding molecular markers. The gene encoding the leucine-rich-repeat-containing G-protein-coupled receptor 5 (*Lgr5*) was first identified as a marker for actively cycling stem cells [4]. The B lymphoma Moloney murine leukemia virus (Mo-MLV) insertion region 1 homolog (*Bmi1*) and the mouse telomerase reverse transcriptase (*mTert*) are specific markers for slowly cycling (also known as dormant or quiescent) intestinal stem cells [5, 6]. Each cell exists at its own position [7] and plays a role. Dormant stem cells can replenish the cycling fraction after severe loss of cycling stem cells. ‘Stemness’ is characterized by the ability to self-renew, proliferate and differentiate. The stemness of intestinal stem cells enables them to reconstitute in the crypts, thus maintaining the cell population over time.

In 2009, an organoid model of intestinal crypts was established using murine Lgr5-EGFP^+^ stem cells [8]. The organoids have a crypt-like structure, and are comprised of all types of epithelial cells [8]. Organoid-forming efficiency (OFE) has been used as an index of the stemness of intestinal cells because stem cells can self-renew, proliferate and differentiate to form organoids with crypt-villus structures. The OFE depends on the number of plated stem cells per well, because stem cells in contact with each other form one organoid in each well [9]. To evaluate the OFE for single stem cells with more precision, we established an efficient ‘one cell/well’ sorting method, with fluorescence-activated cell sorting of single Lgr5-EGFP^high^ cells directly placed into single wells to obtain an organoid. In this context, we defined organoid-forming potential (OFP) as an index for the percentage of organoids formed per plated cell, when one cell was sorted into one well. Based on this index, we confirmed that OFP correlated with the expression of the Lgr5-EGFP gene [9]. In the present study, to clarify the dose and dose-rate response of the stem cell population, we evaluated the abundance of Lgr5-EGFP^high^ stem cells in the intestinal cells and the stemness characterized by OFP.

## MATERIALS AND METHODS

### Mice

Lgr5-EGFP-IRES-Cre^ERT2^ (B6.129P2-Lgr5^tm1(cre/ERT2)Cle^/J; JAX mice #008875) and ROSA26-tdTomato mice (B6.Cg-Gt(ROSA)26Sor^tml4(CAG-tdTomato)Hze^/J; JAX mice #007914) were purchased from Jackson Laboratory (Sacramento, CA, USA). In ROSA26-tdTomato mice, the tdTomato gene is located in the ROSA26 region downstream of a loxP-flanked stop codon sequence. Lgr5-EGFP-IRES-Cre^ERT2^ × ROSA26-tdTomato mice were used in all experiments. The mice were bred in a conventional, clean facility at the Central Research Institute of Electric Power Industry (CRIEPI, Tokyo, Japan), under controlled temperature (24°C ± 2°C) and humidity (45% ± 5%), and exposed to a 12-h light/dark cycle with ad libitum access to γ-sterilized food (CLEA Japan, Tokyo, Japan) and filter-sterilized deionized water. All animal experiments were approved by the Animal Research and Ethics Committee at CRIEPI and performed in accordance with the guidelines for animal care in Japan.

### Lineage tracing

The protocol for the injection of 4-hydroxytamoxifen (4-OHT, Sigma-Aldrich, St. Louis, MO, USA) has been described previously [9]. Briefly, to obtain tdTomato^+^ crypts, 4-OHT was dissolved in sunflower oil at 10 mg/mL and injected intraperitoneally into 6- to 9-week-old mice at 3 mg/40 g body weight.

### Irradiation

For *in vitro* irradiation, immediately after cell sorting, Lgr5-EGFP^high^ cells were irradiated using an MBR-1520R4 X-ray generator (Hitachi Power Solutions, Ibaraki, Japan) operated at 150 kV and 20 mA, and a 0.5 mm Al plus 0.3 mm Cu filter at 0.6 Gy/min (36 Gy/h). For *in vivo* HDR irradiation, the mice were irradiated using a MultiRad 350 generator (Faxitron, Tucson, AZ, USA) operated at 260 kV and 4.5 mA, and a 0.5 mm Al plus 0.3 mm Cu filter at 0.5 Gy/min (30 Gy/h). For *in vivo* LDR irradiation, the mice were irradiated with ^137^Cs at 0.00005 Gy/min (0.003 Gy/h).

### Organoid culture

Tissue preparation was performed as previously described [9]. Briefly, the mice were sacrificed, and the duodenum, jejunum, ileum and colon were harvested and washed in ice-cold phosphate-buffered saline without Ca^2+^ and Mg^2+^ (PBS^−^). Then, intestinal lumen was cut open longitudinally, and the villi of the duodenum, jejunum and ileum, but not the colon, were scraped off using a coverslip. Tissue was cut into 2–3 mm fragments and suspended in PBS^−^ containing 2% fetal bovine serum, washed once in PBS^−^ and incubated in 50 mM EDTA/PBS^−^ for 30 min at 4°C (or on ice) on a rocking platform to separate the crypts from the intestinal tissue. The crypts were passed through a 70-μm cell strainer, washed once with ice-cold PBS^−^ and treated with TrypLE Express (Life Technologies) containing YTC (10 μM Y-27632 (Sigma-Aldrich), 2 μM Thiazovivin and 2.5 μM CHIR99021 (ReproCELL, Kanagawa, Japan)) for 30 min at 37°C to separate cell–cell contacts. To prepare a single-cell suspension, the separated cells were passed through 40- and 20-μm strainers, and then resuspended in PBS^−^ containing 2% fetal bovine serum and YTC. To exclude dead cells, the samples were stained with Fixable Viability Dye eFluor 450 (#65–0863-14, Thermo Fisher Scientific). The cells were isolated using a MoFlo Astrios EQ cell sorter (Beckman Coulter, Brea, CA, USA). Flow cytometry data were analyzed using Summit Software (Beckman Coulter). The isolated individual stem cells were cultured in IntestiCult Organoid Growth Medium (for mouse, Stemcell Technologies, Vancouver, Canada) containing 1× penicillin/streptomycin, 1× N2, 1× B27, 50 ng/mL murine epidermal growth factor (Life Technologies, Carlsbad, CA, USA), 1 mM N-acetylcysteine (Sigma-Aldrich) and 100 ng/mL murine Noggin (PeproTech, Rehovot, Israel). Both YTC and Matrigel (Corning Inc., Corning, NY, USA) were added to the medium immediately before dispensing an individual cell into each well of a flat-bottomed culture plate. To evaluate the OFE, stem cells were sorted directly into 10% Matrigel-containing medium. To evaluate the OFP using by the ‘one cell/well’ sorting method, stem cells were sorted into 1% Matrigel-containing medium in V-shaped wells.

## RESULTS

### Proportion of Lgr5-EGFP ^high^ stem cells and OFE depends on the intestinal region

**Fig. 1.**
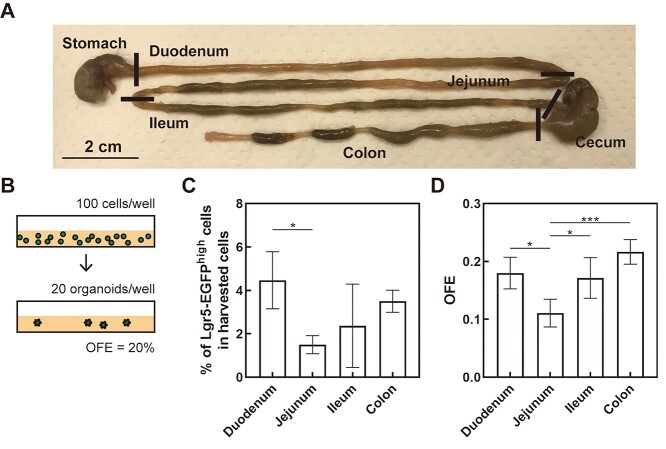
Proportion of Lgr5-EGFP^high^ stem cells in each intestinal region, and their corresponding OFE. (A) Dissected intestines with lines demarcating the intestinal segments. One-third sections of the small intestine were excised and sampled as the duodenum, jejunum and ileum (B) Schematic representation of OFE calculation. (C) Percentage of Lgr5-EGFP^high^ stem cells in the harvested crypt cells. Data excludes doublet cells based on the side and forward scatter values. (D) OFE on culture days 7–8. (C, D) Data represents mean ± SD of groups of four male mice aged 21 ± 5 weeks without 4-OHT injection. ^*^  *P* < 0.05, ^*^^*^^*^  *P* < 0.001 according to the one-way ANOVA with Tukey’s post hoc test.

First, the duodenum, ileum, jejunum and colon (with rectum) were sampled to evaluate whether stem-cell abundance and stemness differ depending on the intestinal region (Fig. 1A). To quantify the stem-cell proportion, the percentage of Lgr5-EGFP^high^ stem cells in the harvested crypt cells was evaluated by flow cytometry. OFE was evaluated as shown in Fig. 1B. The proportion of Lgr5-EGFP^high^ stem cells (Fig. 1C) and OFE (Fig. 1D) varied depending on the intestinal region. To examine whether the proportion and OFE of Lgr5-EGFP^high^ stem cells was affected by irradiation, we compared the colon, including the rectum, with the duodenum, which served as the representative of the small intestine because the number of Lgr5-EGFP^high^ stem cells in the duodenum was higher than that in the ileum or jejunum.

### Only high-dose or high-dose-rate irradiation enhances the replenishment rate of Lgr5 ^+^ stem cells in the duodenum and colon

**Fig. 2.**
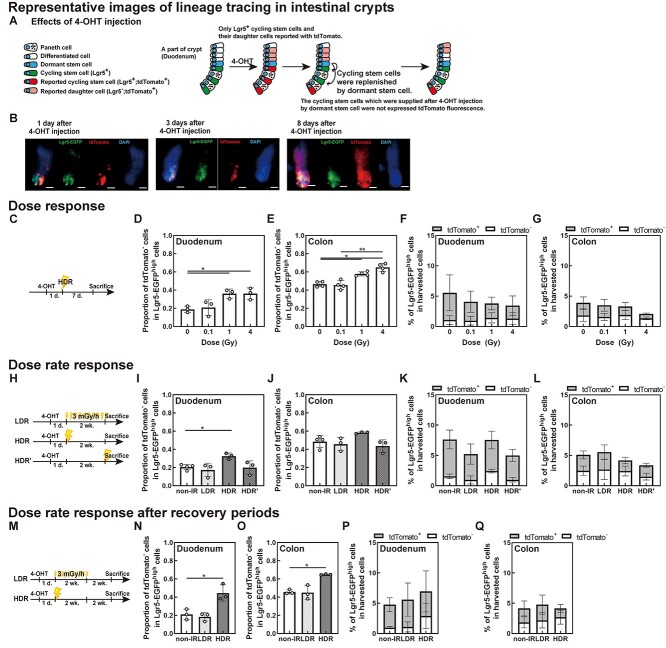
Response of stem cell populations *in vivo* to irradiation doses and dose rates. (A) Effects of 4-OHT injection and lineage tracing. (B) Representative images of duodenum crypts of mice sacrificed 1, 3 or 8 days after 4-OHT injection. White bars: 20 μm. (C–G) Dose response at HDR irradiation. (H–L) Dose rate response. The total dose was 1 Gy. (M–Q) Dose rate response to evaluate the response of low dose-rate irradiation after recovery periods. The total dose was 1 Gy. (C, H, M) Schematic representation of the 4-OHT injection protocol and the irradiation timing. (D, E, I, J, N, O) The proportion of tdTomato^−^ stem cells in Lgr5-EGFP^high^ stem cells in the duodenum (D, I, N) and colon (E, J, O). (F, G, K, L, P, Q) The percentage of Lgr5-EGFP^high^ stem cells in the total number of cells harvested from the duodenum (F, K, P) and colon (G, L, Q). (D–G, I–L, N–Q) Data exclude doublet cells based on the side and forward scatter values. ^*^  *P* < 0.05, ^*^^*^  *P* < 0.01 (D, E, N, O: Repeated measures ANOVA and Tukey’s multiple comparisons test, I, J: Mixed-effects analysis and Tukey’s test, F, G, K, L, P, Q: two-way ANOVA and Sidak’s multiple comparisons test).

Genetic lineage-tracing is applicable to evaluate the replenishment rate of cycling stem cells originating from dormant cells, and the number of cycling stem cells after irradiation. After 4-OHT injection, Lgr5-EGFP^+^ stem cells exhibited tdTomato expression via tamoxifen-driven Cre–LoxP recombination (Fig. 2A and B). The expression of tdTomato was exclusive to Lgr5^+^ stem cells and their daughter cells (Fig. 2A). Thus, the dormant stem cells or the differentiated cells and cycling stem cells, which replenished the dormant stem cells after 4-OHT injection, did not express tdTomato. The time course of 4-OHT injection and irradiation are shown in Fig. 2C, H and M. The proportion of tdTomato^−^ stem cells in the Lgr5-EGFP^high^ stem cells increased significantly with 1 and 4 Gy doses, but not with 0.1 Gy, in both duodenum (Fig. 2D) and colon (Fig. 2E) irradiated at HDR. The percentage of Lgr5-EGFP^high^ stem cells in the cells harvested from the intestines tended to decrease with increasing doses at 1 week after irradiation (Fig. 2F and G). tdTomato^−^;Lgr5-EGFP^−^ cells are non-stem cells derived from Lgr5^+^ stem cells, although tdTomato^−^;Lgr5-EGFP^−^ cells included the stem cells without Lgr5-EGFP expression. The proportion of all tdTomato^+^ cells and that of tdTomato^+^;Lgr5-EGFP^−^ daughter cells in irradiated mice showed no significant differences compared with those in the control group (Supplementary Fig. S1).

**Fig. 3.**
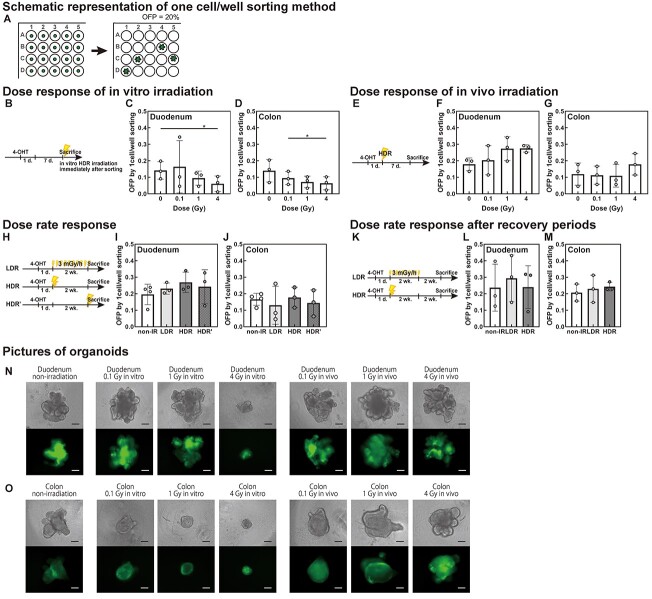
Response to dose and dose-rate of irradiation over time assessed by measuring OFP. (A) Schematic representation of one cell/well sorting and an example of OFP, where 20 organoids formed in a 96-well plate, resulting in an OPF of 20.1%. (B–D) Dose response of *in vitro* HDR irradiation. (E–G) Dose response of *in vivo* HDR irradiation. (H–J) Dose rate response. Total dose was 1 Gy. (K–M) Dose rate response to evaluate the response of low dose-rate irradiation after recovery periods. The total dose was 1 Gy. (B, E, H, K) Schematic representation of the 4-OHT injection protocol and irradiation timing. For irradiation *in vitro*, stem cells were irradiated immediately after sorting (B). For irradiation *in vivo*, mice were irradiated 7 days before sacrifice (E). (C, D, F, G, I, J, L, M) OFP determined after 1 cell/well sorting. Data represent mean ± SD of three or four mice aged around 9 weeks at sacrifice. (^*^  *P* < 0.05, Repeated measures ANOVA and Tukey’s multiple comparisons test). (N, O) Micrographs of duodenal (N) and colonic organoids (O) on days 12–14. Upper micrographs show phase-contrast and lower images show Lgr5-EGFP fluorescence. Scale bar: 100 μm.

The proportion of tdTomato^−^ stem cells in the Lgr5-EGFP^high^ stem cells did not change immediately after irradiation with 1 Gy at HDR, but increased after 1 week (Supplementary Fig. S2A and B). The percentage of Lgr5-EGFP^high^ stem cells tended to decrease immediately and at 1 week after irradiation, but recovered at 2 weeks and 4 weeks after HDR irradiation (Supplementary Fig. S2C and D).

The proportion of tdTomato^−^ stem cells in the Lgr5-EGFP^high^ stem cells did not increase after irradiation with 1 Gy at LDR (Fig. 2I, J, N and O), even if the mice were allowed to recover for 2 weeks (Fig. 2N and O). In contrast, the percentage of Lgr5-EGFP^high^ cells decreased immediately after LDR irradiation (Fig. 2K), but recovered after 2 weeks in the duodenum (Fig. 2P). In the colon, there were no LDR irradiation-induced changes (Fig. 2L and Q). These trends were the same for 3 mGy/h irradiation and 1.5 mGy/h irradiation (Supplementary Fig. S3).

### Assessing organoid-forming potential to determine response to irradiation

OFP was evaluated following cell sorting using our ‘one cell/well’ [9] method, as schematically presented in Fig. 3A. The Lgr5-EGFP^high^ stem cells were irradiated *in vitro* immediately after one cell/well sorting to evaluate the effect of irradiation on each stem cell (Fig. 3B). To evaluate the effects on the intestine *in vivo*, the mice were irradiated 1 day after 4-OHT injection (Fig. 3E, H and K), and the Lgr5-EGFP^high^ stem cells were identified by tdTomato expression at the time of irradiation (Fig. 2A and B).

When the Lgr5-EGFP^high^ stem cells were irradiated *in vitro* immediately after plating, OFP decreased significantly with increasing doses at HDR (Fig. 3C and D). The OFP of Lgr5-EGFP^high^ stem cells irradiated *in vivo* tended to be higher than that of non-irradiated intestines (Fig. 3F and GK). There was no significant difference in OFP between LDR and HDR irradiation with a total dose of 1 Gy in both duodenum and colon (Fig. 3I, J, L and M). In addition, the proportion of tdTomato^+^ stem cells in the Lgr5-EGFP^high^ stem cells and the percentage of the Lgr5-EGFP^high^ stem cells in the harvested crypt cells were not significantly different between LDR and HDR irradiation (Fig. 2). Representative images of organoids are shown in Fig. 3N and O. The 4-Gy-irradiated organoids *in vitro* did not grow well, or form a budding structure, meanwhile irradiated organoids *in vivo* grew normally, like non-irradiated organoids.

## DISCUSSION

Radiation-induced apoptosis of stem cells is well known in intestine [10, 11]. In this study, the proportion of Lgr5-EGFP^high^ stem cells was decreased compared with control by irradiation. The proportion of tdTomato^−^ in the Lgr5-EGFP^high^ stem cells was increased by 1 and 4 Gy irradiation at HDR but not by 0.1 Gy irradiation at HDR or 1 Gy irradiation at LDR. The proportion of tdTomato^−^ in the Lgr5-EGFP^high^ stem cells plateaued above 1 Gy probably because the number of stem cells in a duodenum crypt is large and the stem cell population is heterogeneous of radiation sensitivity. The proportion of Lgr5-EGFP^high^ tdTomato^−^ cells in the harvested crypt cells did not decrease with HDR irradiation but that of Lgr5-EGFP^high^ tdTomato^+^ cells tended to decrease. This suggests that the number of stem cells decreased with irradiation, and then showed an increase in the rate of replenishment. Increased replenishment may contribute to increased intrinsic mutation due to cell division. The proportion of tdTomato^−^ stem cells in the Lgr5-EGFP^high^ stem cells did not increase immediately after irradiation probably because cell repair and proliferation require some time. The proportion of tdTomato^−^ stem cells after irradiation with 1 Gy at HDR increased with time, without reproducing the tdTomato^+^ stem cells. Consequently, the Lgr5-EGFP^high^ stem cells, newly replenished by dormant stem cells, can occupy the crypts instead of the HDR-irradiated Lgr5-EGFP^high^ stem cells, probably as a result of stem cell competition. In general, dormant stem cells are less damaged by mutagenesis than cycling stem cells [12, 13]. Conversely, the proportion of tdTomato^−^ cells did not change with LDR, although the proportion of stem cells decreased after 1 Gy irradiation at LDR.

The OFE of the crypt-base columnar cells or surviving fractions of organoid decreases with increasing *in vitro* HDR irradiation dose [14, 15]. In the present study, individual stem cells were cultured in a medium that mimicked the *in vivo* environment [9] although stem cells in this model system are not affected by niche cells, such as Paneth cells. We observed that OFP decreased with an increasing irradiation dose *in vitro*. This observation suggests that the cycling stem cells died or lost stemness depending on the dose. However, the tdTomato^+^ Lgr5-EGFP^high^ stem cells, which maintained Lgr5-EGFP expression after HD irradiation *in vivo,* had a relatively high OFP. Importantly, tdTomato^+^ Lgr5-EGFP^high^ cells were the cycling stem cells that were irradiated and maintained Lgr5-EGFP expression because the mice were injected with 4-OHT and then irradiated. This means that some tdTomato^−^ cycling stem cells were newly supplied by dormant cells after irradiation. The differences between *in vitro* and *in vivo* irradiation may arise from whether the surrounding cells can support each other *in vivo*. Damaged stem cells, which could not form organoids, were eliminated from the cell population for 1 week after irradiation, and the remaining tdTomato^+^ Lgr5-EGFP^high^ stem cells maintained stemness after irradiation, similar to non-irradiated stem cells.

The cancer risk due to LDR is lower than that due to HDR irradiation even when the cumulative doses are the same [16, 17]. This observation has been described as the ‘dose-rate effect’ [1], but the mechanisms underlying the dose-rate effect have not yet been fully clarified. The International Commission on Radiological Protection (ICRP) Publication 131 postulated that cell competition, which is generally observed as neutral competition, could explain the dose-rate effect [12]. Accordingly, extreme LDR irradiation, where irradiated and non-irradiated cells coexist in the cell population, causes the irradiated cells to be eliminated more easily from the population than non-irradiated cells [12]. Thus, the LDR-irradiated population will remain as healthy as the non-irradiated cell population. Previously, we described the possibility of radiation-induced stem-cell competition using duodenal organoids [9]. In the present study, we focused on the stemness and stem-cell population.

After HD irradiation at HDR, the cycling stem cells apparently lost Lgr5-EGFP expression, and their stemness was enhanced probably because of the higher OFP of the cycling stem cells maintaining Lgr5-EGFP expression. However, after 1 Gy irradiation at LDR, the proportion of tdTomato^−^ in the Lgr5-EGFP^high^ stem cells and OFP was the same as that in non-irradiated cells, whereas the proportion of stem cells decreased slightly. This suggests that the replenishment by dormant cells, stemness of surviving Lgr5^+^ cells and stem-cell competition are all enhanced after 1 Gy HDR irradiation, whereas only stem-cell competition is induced under LDR condition.

Interestingly, 0.1 Gy irradiation at HDR and 1 Gy irradiation at LDR showed that the proportion of tdTomato^−^ cells in Lgr5-EGFP^high^ cells was not different in the control and % of Lgr5-EGFP^high^ cells in harvested cells tended to decrease. There is a possibility that cells do not have to proliferate rapidly to fill the vacancy caused by radiation damage, and healthy cells can be selected by cell competition; this is an interesting topic for future studies. In the Lgr5-EGFP^high^ stem cells damaged by 0.1 Gy HDR or 1 Gy LDR irradiation, the more damaged cells may be eliminated from the stem cell population by radiation-induced stem-cell competition, because all cells were not damaged uniformly. For example, if the mean number of double-strand breaks (DSB) in cells treated with 1 Gy is 40, the percentage of the cells with DSB of 20 or less is 0.04% for 1 Gy irradiation, because the radiation attack on cells follows the Poisson distribution. Similarly, 1.8% of the cells do not have DSB, 7.3% have only one DSB, 19.5% have four DSBs and 2.1% cells have nine or more DSBs in a 0.1 Gy-irradiated cell population. The proportion of serious events after 0.1 Gy irradiation is different from that caused by high doses. Thus, the stem-cell population may remain healthy because the Lgr5^+^ stem cells, which are seriously damaged, easily lose in the stem cell competition. Our previous study indicated the possibility that radiation-induced competition could explain the dose-rate effect [9]. Our results support that radiation-induced competition is important after irradiation at LDR and LD/HD irradiation at HDR. The expression of p53 increases after irradiation [18], and decides the cell-cycle fate of daughter cells [19]. Thus, the degree of damage from irradiation could potentially relate to the fitness that determines the outcomes of cell competition.

Overall, the kinetic response of stem cells to radiation differed in cells receiving 0.1 Gy and 1 Gy HDR irradiation. Above 1 Gy, excessive division due to compensatory growth may result in mutations. In contrast to HDs of HDR irradiation, the stem cells would have enough time to repair and compete at LDs of HDR and HDs of LDR. Our results support the notion that the radiation risk due to HDs should not apply directly to LDs (< 100 mGy) and that the risk due to HDRs should not apply directly to LDRs.

The mechanisms underlying the differences in carcinogenesis between small and large intestinal stem cells have not been elucidated. A few studies have reported the effects of radiation on the stem cells of the large intestine [15, 18, 20, 21]. When radiation-induced DSBs were studied in the gene encoding p53-binding protein 1 (*53BP1*), no significant differences were found between the colon and duodenum [18]. The number of stem cells in the small intestine is two- or three-fold higher than that in the large intestine [20]. The number of colonic Lgr5^+^ stem cells significantly decreases after exposure to 1 Gy HDR radiation [11]. However, the decrease tended to be less in the colon than in the duodenum (Fig. 2). Thus, replenishment was enhanced in the duodenum compared with that in the colon (Fig. 2). The OFP increase in the large intestine with increasing doses *in vivo* was smaller than that in the duodenum (Fig. 3). The cell cycle is reportedly longer in the large intestine [20], and irradiation affects the large intestine more than the duodenum [21]. Thus, the large intestine may more easily accumulate radiation effects than other regions of the intestine.

## Supplementary Material

20210921_supplement_final_rrab120
