# Supplementary material for: Ionizing radiation alters organoid forming potential and replenishment rate in a dose/dose-rate dependent manner
Source: J Radiat Res. 2021 Dec 31;63(2):166–73. doi: 10.1093/jrr/rrab120 (PMC8944312; doi:10.1093/jrr/rrab120)
Supplement: 20210921_supplement_final_rrab120 [file 20210921_supplement_final_rrab120.docx]

**SUPPLEMENTARY DATA**

**
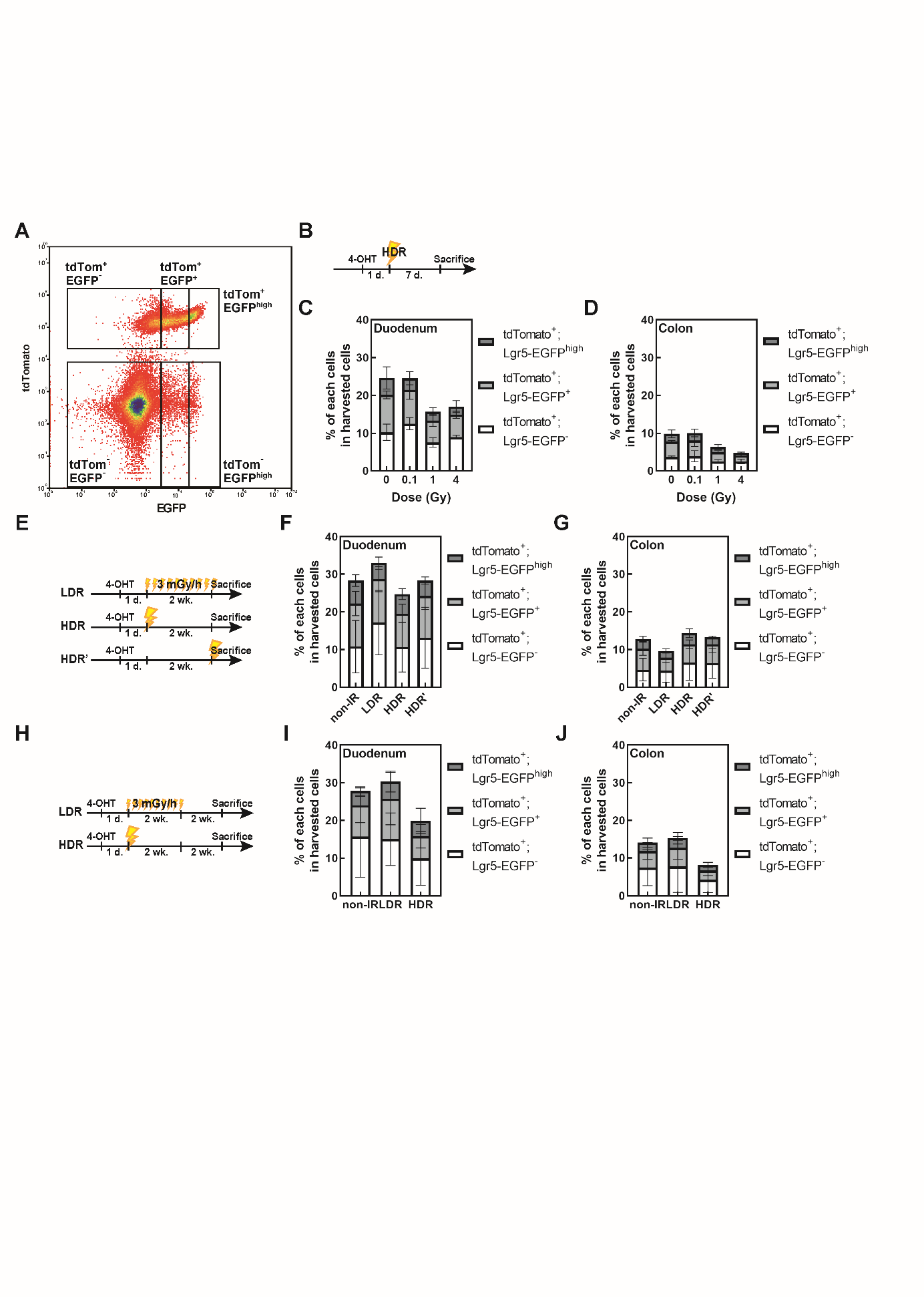
**

Figure S1 Proportion of tdTomato^+^;Lgr5-EGFP^-^ daughter cells

(A) The gate of EGFP and tdTomato (tdTom). (B–D) Dose response of the duodenum (C) and colon (D). (E–G) Dose rate response of the duodenum (F) and colon (G) in the case of 2 weeks between 4-OHT injection and sacrifice. (H–I) Dose rate response of the duodenum (I) and colon (J) in the case of 4 weeks between 4-OHT injection and sacrifice. (C, D, F, G, I ,J) Data exclude doublet cells based on the side and forward scatter values. * *P* < 0.05, ** *P* < 0.01, *** *P* < 0.001 according to the two-way ANOVA and Tukey’s multiple comparisons test. (C) ** 0.1 Gy vs. 1 Gy of tdTomato^+^;Lgr5-EGFP^-^, * 0 Gy vs. 1 Gy and 0 Gy vs. 4 Gy of tdTomato^+^;Lgr5-EGFP^+^. (D) *** 0 Gy vs. 4 Gy and 0.1 Gy vs.4 Gy, * 0 Gy vs. 1 Gy and 0.1 Gy vs. 1 Gy of tdTomato^+^;Lgr5-EGFP^+^.


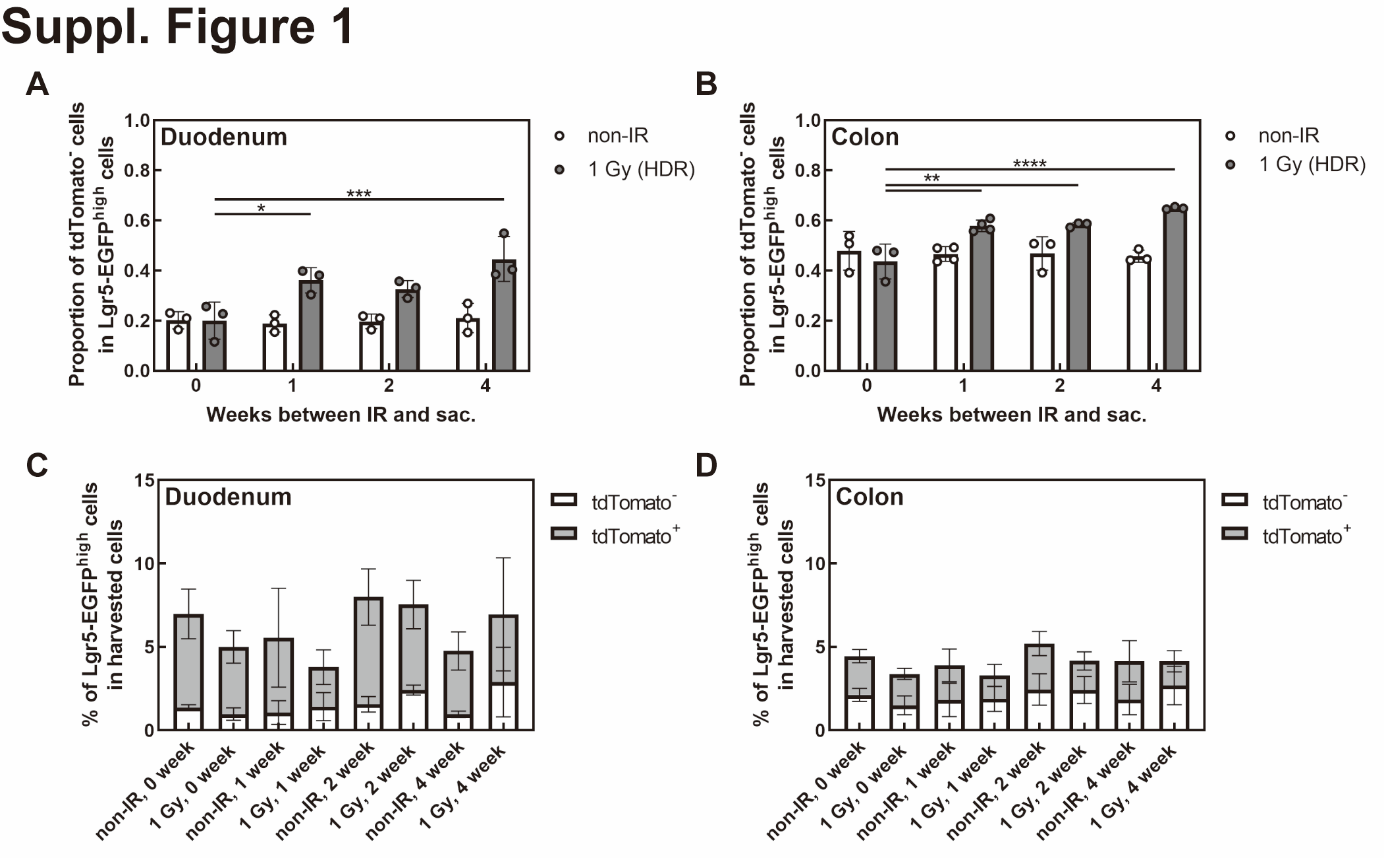


Figure S2 Response to irradiation over time

(A, B) The proportion of tdTomato^−^ stem cells in Lgr5-EGFP^high^ stem cells. (C, D) The percentage of Lgr5-EGFP^high^ stem cells in the total number of harvested cells for non-IR and 1 Gy treatments, each at different weeks after irradiation. (A, C) Duodenum. (B, D) Colon. (A-D) Data at 0 weeks after irradiation for HDR’ and non-IR cells in Figs. 2I–L, data at 1 week after irradiation for Figs. 2D–G, data at 2 weeks after irradiation for HDR and non-IR in Figs. 2I–L, and data at 4 weeks after irradiation for HDR and non-IR in Figs. 2N–Q. non-IR: non irradiated samples, HDR: high dose rate. * *P* < 0.05, ** *P* < 0.01, *** *P* < 0.001, **** *P* < 0.0001 (two-way ANOVA with Sidak’s multiple comparisons test).


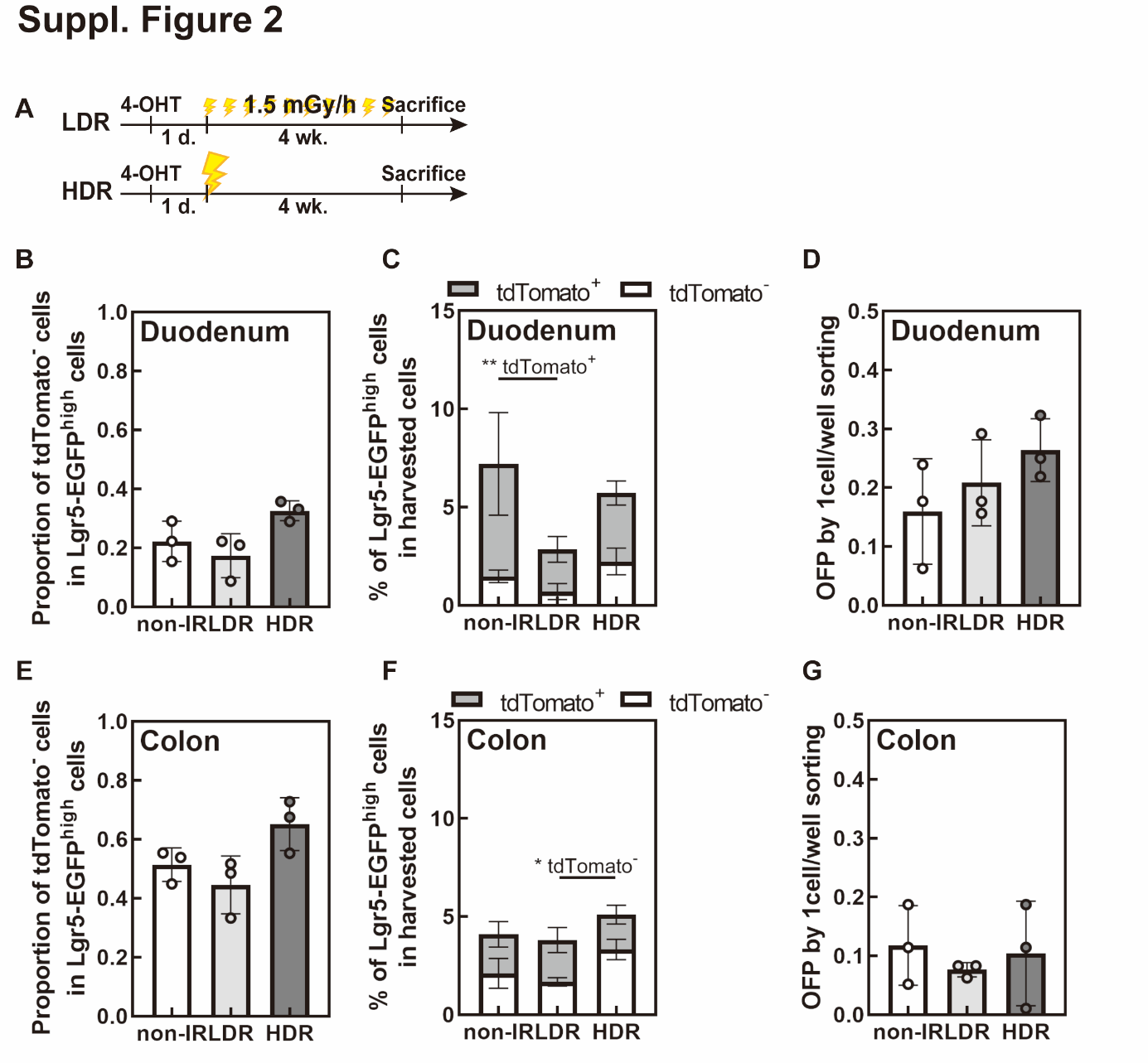


Figure S3 Effects of low-dose-rate irradiation

(A) Schematic representation of the 4-OHT injection protocol and irradiation timing. The mice were irradiated with ^137^Cs at 0.000025 Gy/min (0.0015 Gy/h). The total dose was 1 Gy. (B– D) Duodenum. (E–G) Colon. (B, E) The proportion of tdTomato^−^ stem cells in Lgr5-EGFP^high^ stem cells. (C, F) The percentage of Lgr5-EGFP^high^ stem cells in the total number of harvested cells. (B, C, E, F) Data exclude doublet cells based on the side and forward scatter values. (D, G) The organoid-forming potential (OFP) assessed by 1 cell/well sorting. * *P* < 0.05, ** *P* < 0.01 (B, D, E, G: Repeated measures ANOVA with Tukey’s multiple comparisons test, C, F: two-way ANOVA and Sidak’s multiple comparisons test).
